# Trophic state in a tropical lake based on Chlorophyll‐a profiler data and Sentinel‐2 images: The onset of an algal bloom event

**DOI:** 10.1002/wer.1590

**Published:** 2021-06-22

**Authors:** Diego A. Pantoja, Néstor A. Vega‐Álvarez, Tzitlali Gasca‐Ortiz

**Affiliations:** ^1^ Department of Physics Universidad de Guadalajara Guadalajara Mexico; ^2^ Faculty of Physics and Mathematics Universidad Michoacana de San Nicolás de Hidalgo Morelia Mexico

**Keywords:** chlorophyll‐a indices, deep and high‐mountain lake, eutrophication indices, Lake Zirahuén, Mexican lake, small, spectral reflectance

## Abstract

The condition of Lake Zirahuén was evaluated before and after a significant algal bloom took place. The state of the lake was tracked using measurements of chlorophyll‐a concentration from a fluorometer attached to a conductivity, temperature, and depth (CTD) profiler and complemented with the use of satellite images from Sentinel‐2. It was found that the current state of the lake is in transition from an oligotrophic to a mesotrophic level with some evidence of deterioration, but still being considered as a clear lake. With the use of empirical algorithms to retrieve chlorophyll‐a from satellite data it was found that the images presented an acceptable feasibility (*R* ^2^ = 0.64). The observed algal bloom was detected one month before the algae reached its major extent, being the reflectiveness of the green band a good indicator of the onset of the algal bloom event. Favorable conditions for the algal bloom were also recorded two months before its major extend through a chain of thermistors.

**Practitioner Points:**

- The trophic state of Lake Zirahuén was evaluated before and after a significant algal bloom took place.
- The lake is classified as being in transition from oligotrophic to mesotrophic. Nonetheless, it continues to have good clarity.
- Satellite images improve in the description of the spatial‐temporal variability of the lake. In particular, the green band reflectiveness.

## Graphical abstract

The presence and detection of an algal bloom event.

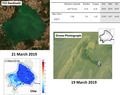

## INTRODUCTION

Analyzing lakes based on the amount of nutrients reaching their basin is of great importance because the proper management and conservation is vital due to the high impact this has on the lake's ecological and social environment (Carlson, 1977; Gibson et al., 2000; Harper, 1992). Also, because lakes are one of the most important habitats and food resources for a wide variety of fish, aquatic life, and wildlife; in addition to its importance as a sources of water for human consumption (Boyd, 2015; Wetzel, 2001). However, although there are some processes that occur naturally in any lake that can be beneficial for all kinds of life, there are other issues such as eutrophication, which is well known to be not desirable.

In lakes, the concentration of chlorophyll‐a (Chl‐a) is widely used to measure the biomass of phytoplankton, and hence, the concentration of nutrients, that is, the Chl‐a is a common biological indicator of eutrophication (Felip & Catalan, 2000; Simpson & Sharples, 2012; Wetzel, 2001). For lakes near human settlements, eutrophication process is inevitable if inadequate and inefficient management is applied. For example, contamination can occur due to runoff of wastewater, detergents, and other kinds of organic matter that enter the aquatic ecosystem from human settlements. Furthermore, if the lake is located near a rural zone in which agricultural practices are among the primary economic activities, an extra stressor is added with a highly negative impact (de‐Anda & Shear, 2001). Climate change is another factor that influence on water eutrophication due to the occurrence of extreme events in water bodies. This is an important factor that has immediate impact on the lake state since this could cause an extreme increase/decrease in water temperature (Adrian et al., 2009). If temperature increases, this promotes stronger vertical thermal gradients which results in less vertical interchange during spring/summer in tropical lakes, on the contrary if temperature decreases, this promotes stronger vertical mixing during winter which can result in the interchange of all kinds of sediments in the water column. If both events happen, first an extreme vertical mixing and then a stronger vertical thermal gradient, these are the perfect ingredients to develop an algal bloom.

Different methods or indices exist to determine the trophic state of a lake (Carlson, 1977; Gibson et al., 2000). In agreement with the classification of the OECD (Organisation for Economic Cooperation & Development, 1982), the degree of eutrophication of a lake is defined according to three parameters: (i) the concentration of total phosphorus, (ii) the concentration of Chl‐a, and (iii) the transparency. The conventional limit values for this criterion are shown in Table 1.

**TABLE 1:** Limit values for trophic classification according to the OECD, where Pt is the total phosphorus concentration and SD is the depth of the Secchi disk

Trophic classification	P*t* (*μ*g/L)	Chlorophyll‐a (*μ*g/L)		Transparency SD (m)	
		Medium	Maximum	Medium	Maximum
Ultra‐oligotrophic	˂4.0	˂1.0	˂2.5	˃6.0	˃12.0
Oligotrophic	˂10.0	˂2.5	˂8.0	˃6.0	˃3.0
Mesotrophic	10–35	2.5–8.0	8.0–25	6.0–3.0	3.0–1.5
Eutrophic	35–100	25–75	25–75	3.0–1.5	1.5–0.7
Hypertrophic	˃100	˃75	˃75	˂1.5	˂0.7

Notably, an adequate classification can be established using only the measurements of Chl‐a. Although this situation could be a limitation for making a more accurate classification, it is also quite practical to establish the trophic state with information from a single parameter, despite the complex interaction between the numerous variables involved in the eutrophication process (Janus & Vollenweider, 1981).

**FIGURE 1:**
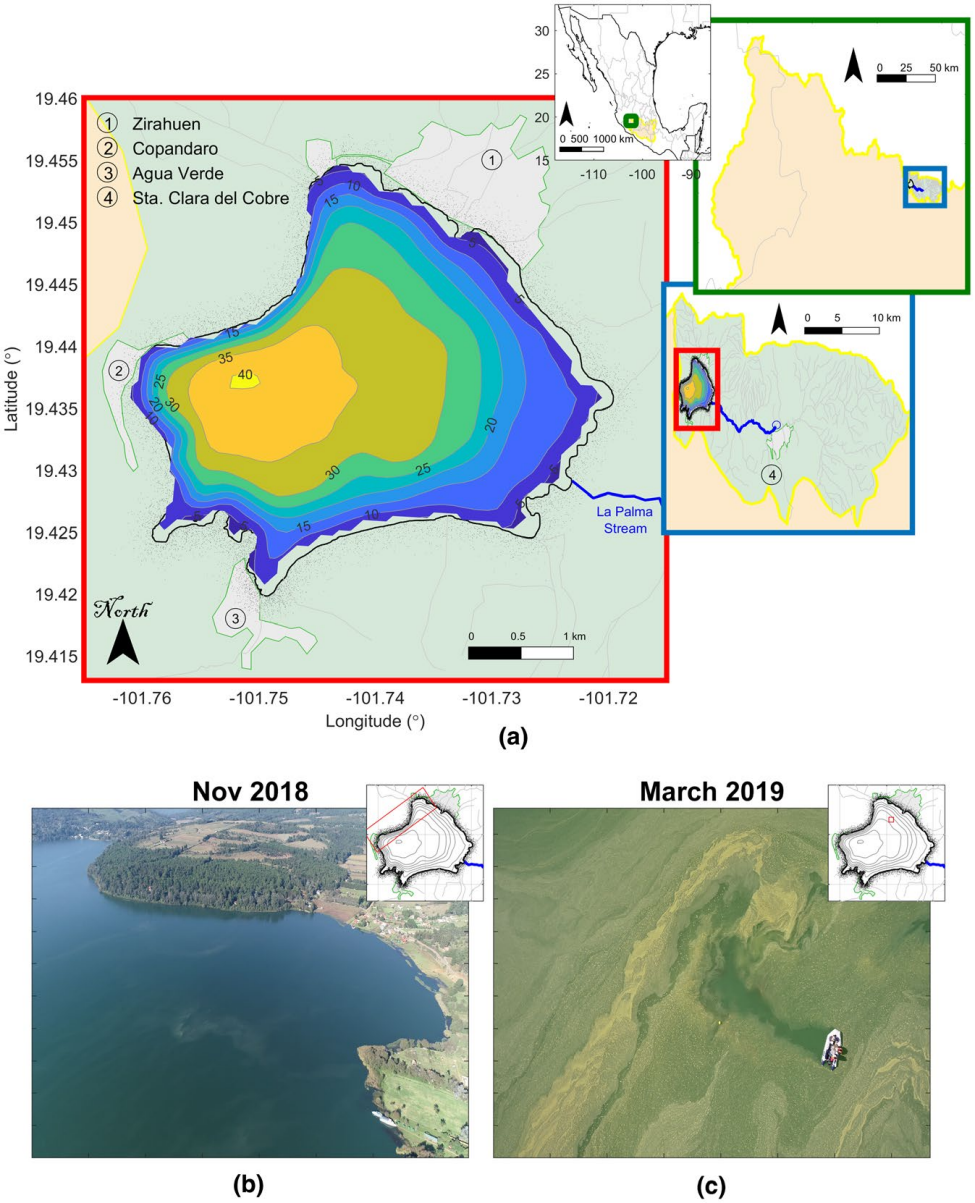
(a) Study area of lake Zirahuén. Color contours in the lake correspond to bathymetric isolines, marked every 5 m. Circled numbers correspond to local communities at the rim of the lake, with the exception of no. 4 which is connected to the lake through the La Palma stream. The insets show the location of the Balsas basin (cream color) and Tepalcatepec subbasin (light green color) in México. Gray lines correspond to secondary streams. To show contrasting color of the appearance of the lake, in (b) drone photograph of Lake Zirahuén on 23 November 2018. The red rectangle in the inset shows the perspective of the photograph, in (c) drone photograph of Lake Zirahuén on 19 March 2019, clearly showing an algal bloom. The red rectangle in the inset shows the point of view of the photograph

In Lake Zirahuén, Figure 1, the trophic state and water quality have been previously evaluated. Martínez‐Almeida and Tavera (2005) carried out a hydrological study to interpret the presence of algae in the lake concluding that the lake was classified as oligotrophic according to measurements of Chl‐a. Rendón et al., (2010) and Vergara de Paz et al., (2010) carried out some studies based on measurements of biogeochemical parameters, dynamics of nutrients and various trophic indices in which it was concluded that the lake is in transition from oligotrophic to mesotrophic. In addition, the authors commented that the lake has remained transparent, although being affected by agricultural and industrial‐artisanal activities and domestic water discharge. Mendoza et al., (2015) found that the water quality conditions are acceptable for the preservation of aquatic life and argued that livestock grazing, and land use or land cover change could affect the trophic state of the lake if not controlled in time. It was also found that the lake water does not meet drinking water standards and must be purified prior to drinking. Notably, all of the communities on the lake shore have a piped water supply but do not have a public drainage network adding an extra source of infiltration issues due to the long‐standing use of latrines (INEGI, 2010).

Lake Zirahuén is a source of food and provides some income for the residents of the rural population living on the lake´s shore because it attracts tourism to the area and enables fishing activity. However, the primary economic activities in the region are the agriculture, animal grazing, and wood industries (Ortiz Paniagua & Rendón López, 2010), followed by fishing, craftsmanship, commerce, and tourism.

Unfortunately, Lake Zirahuén was affected by an algal bloom during the early year of 2019, which increased the turbidity of the water near the surface layer, Figure 1c. Thus, communities around the shore became concerned about the yellowish layer that appeared to increase the level of water contamination due to the unusual increase in algae that occurred at this time and during several other episodes of the previous decade (Ortiz Paniagua & Rendón López, 2010).

Then this study will focus on in situ observations combined with satellite data that can be used to improve the monitoring of the lake in time and space, in particular, with the use of reflectiveness values it is expected that the green band reflectivity of satellite sensors will be used to track regularly the state of the lake. So, the aim of this study is to investigate if it is possible to determine whether or not a eutrophication process is being developed, a result that could be useful to raise the hypothesis that in future strategies this methodology can be implemented to monitor the problem of eutrophication in Lake Zirahuén.

To our knowledge, this is the first study of this type of Lake Zirahuén, México, a small, deep, and high‐mountain lake in which an algal bloom, most likely caused by agricultural activities in the surrounding rural zone, was identified. This also will serve as evidence of the deterioration suffered by the lake in recent years, both naturally and due to anthropogenic causes, accelerated by sewage discharge, the daily washing of clothes with detergent on the shore of the lake, and the recent change of land use in the watershed (Bernal‐Brooks et al., 2002; Ortiz Paniagua & Rendón López, 2010).

### Study area

Lake Zirahuén is located in the south‐central portion of Mexico, within the hydrological region of the Balsas basin, between coordinates 19°21'10”–19°29'24” N and 101°29'37”–101°49′37″ W; Figure 1a. The lake is in the western region of the basin (Tepalcatepec subbasin) at 2080 m above sea level and has an area of 10.48 km^2^ and a length of between 3.5 and 4.5 km. It is a monomictic lake, currently considered to be oligo‐mesotrophic, of endorheic type, with a maximum depth of about 40 m. The lake is stratified in summer, when it reaches a surface temperature of around 24℃, and mixed during winter, when it reaches a temperature around 16℃ through the entire water column. The predominant climate is C(w2) according to the Köppen classification (García, 2004), that is, a temperate sub‐humid climate with rain during summer and an average annual temperature ranging between 12 and 18℃. The average annual precipitation is 900 mm. It has only one entrance, “La Palma” stream (also known as “El Silencio” stream) in the eastern part of the lake with a flow rate of less than 0.5 m^3^/s (Bernal‐Brooks et al., 2002; Chacon‐Torres & Rosas‐Monge, 1998; Comisión Estatal del Agua y Gestión de Cuencas (CEAGC), 2016; ; ; ; ; ; ; Gasca‐Ortiz et al., 2020; Martínez‐Almeida & Tavera, 2005; Mendoza et al., 2015; Tavera & Martínez‐Almeida, 2005).

## METHODOLOGY

Three campaigns were compared to monitor Lake Zirahuén: (i) November 23, 2018, (ii) March 19, 2019, and (iii) June 19, 2019. That is, before and after an algal bloom took place at the beginning of the year 2019. Also, a thermistors chain and a series of weekly satellite images were used with approximately one year of data.

### CTD data

The water quality and thermal structure of Lake Zirahuén was measured using a CTD (Conductivity, Temperature, and Depth) profiler XR‐620 (RBR Ltd., Ottawa, ON Canada), using additional sensors for Chl‐a. The CTD was dropped manually from a boat acquiring a vertical speed of about 1 m/s and sampling at a rate of 1 Hz. All of the variables were linearly interpolated to smooth values around the thermocline by removing spikes due to sensor issues.

### Thermistor chain

To continuously measure the temperature in the lake, a water thermistors chain was placed with 2 Hobo temperature loggers (Onset Computers Corporation, Bourne, MA, USA) at depths of 26 and 31 m, sampling at a rate of 12 min near the northern part of the lake at a depth of ~32 m. The sample period was from November 22, 2018, to November 18, 2019.

### **Sentinel‐2** **MSI imagery**

Because monitoring a lake is an intricate task that demands time and resources, an excellent alternative procedure for lake monitoring is the remote sensing techniques that have been developed and improved in recent years, particularly the use of satellite sensors (Cairo et al., 2020; Toming et al., 2016). Recently, satellite data has been used to monitor water quality and trophic state based on the concentration of Chl‐a, turbidity, among other parameters. It has been demonstrated that remote sensing is useful for inland water monitoring even due to the relative size of the water body and its location near to cities, woods, or others human settlements. This technique is particularly useful for lakes in which anthropogenic factors have significantly altered the surrounding environment due to increased changes in land use to agricultural and industrial activities (Chacon‐Torres et al., 1992; Grendaitė et al., 2018; Membrillo‐Abad et al., 2016; Toming et al., 2016).

Sentinel‐2 MSI (MultiSpectral Instrument) is a Copernicus program of the European Space Agency (ESA) consisting of two polar‐orbiting satellites: Sentinel‐2A and Sentinel‐2B. The multi‐spectral instrument onboard each satellite is able to recover data in 13 spectral bands at a variable and high resolution of 10, 20, and 60 m within 290 km of swath and with a revisit time of 5 days (European Space Agency (ESA), 2015). These constellations of satellites were launched separately in 2015 and 2017 and have been used in numerous places and applications to improve upon the performance (spatially and/or in terms of radiometric resolution) of their predecessors and contemporaneous satellite sensors: MODIS (Moderate Resolution Imaging Spectroradiometer), MERIS (Medium Resolution Imagining Spectrometer), SPOT (satellite for observation of earth), Landsat satellite series, and, recently, Sentinel‐3 and Sentinel‐4, among others (Chacon‐Torres et al., 1992; Membrillo‐Abad et al., 2016; Soomets et al., 2020; Toming et al., 2016).

#### *Estimation* *of Chl*‐*a and turbidity*

The concentration of Chl‐a and a proxy for turbidity was estimated by applying the MSI imagery from Sentinel‐2 satellite Level 2A products, based on empirical equations that combine reflectance from different bands (Grendaitė et al., 2018; Peppa et al., 2020). The indices used in this study for parameter estimation were (B5‐B6) for Chl‐a and (B4) for turbidity (Cairo et al., 2020; Membrillo‐Abad et al., 2016; Peppa et al., 2020), where B4, B5, and B6 are the bands of reflectance at $\lambda_{B4}=665\;\mathrm{nm}$, $\lambda_{B5}=705\;\mathrm{nm}$, and $\lambda_{B6}=740\;\mathrm{nm}$ central wavelengths, respectively, (see Supplementary materials for the analysis of the accuracy of the estimations). In the case for the Chl‐a, this index weights the height of the peak of the B5 band against the baseline of its adjacent peaks of either the B4 and/or the B6 spectral bands. For turbidity, as an indirect measure of transparency, the index was based on the spectral range of 650–680 nm commonly used to estimate the water clarity of medium to low turbidity waters (Membrillo‐Abad et al., 2016). Although these indices were originally designed for early sensor satellites (e.g., MERIS or SPOT‐5), these empirical equations have been used extensively as a representative indicators of the trophic state of several lakes using Sentinel‐2 MSI data (Grendaitė et al., 2018; Peppa et al., 2020; Toming et al., 2016).

The MSI images used here were cloud free during 2019. There were a total of 73 satellite images, all of which were downloaded using the Copernicus Open Access Hub (European Space Agency (ESA), 2020). By visual inspection, 34 images were cloudless (47%), 9 were partially clouded (12%), and 30 were totally clouded (41%). When possible, a mask was applied to the partially clouded images to remove cloudiness and retrieve proper averaged data from the images. In addition, a mask was applied around the shore of the lake to avoid the interference of macrophytes that are commonly located near the rim of the lake. The depth of the lake also did not cause any interference of the reflectance. The images were taken around 12:00 pm local time (17:00 Z‐time).

## RESULTS

### Thermal structure

For relatively deeper lakes in which a thermal structure forms, the development of a thermocline is vital for the dynamic control of Chl‐a and other biogeochemical substances (Simpson & Sharples, 2012). In general, the thermal structure of Lake Zirahuén shows a typical behavior, with a surface mixed layer forming well during the spring‐summer period, and a totally mixed layer forming during the autumn‐winter period (Gasca‐Ortiz et al., 2020). These thermal structures, combined with the internal processes proper of the lake, result in the characteristic behavior of Chl‐a that is taking place in the lake, as shown below.

#### *CTD*‐*profiler temperature*

**FIGURE 2:**
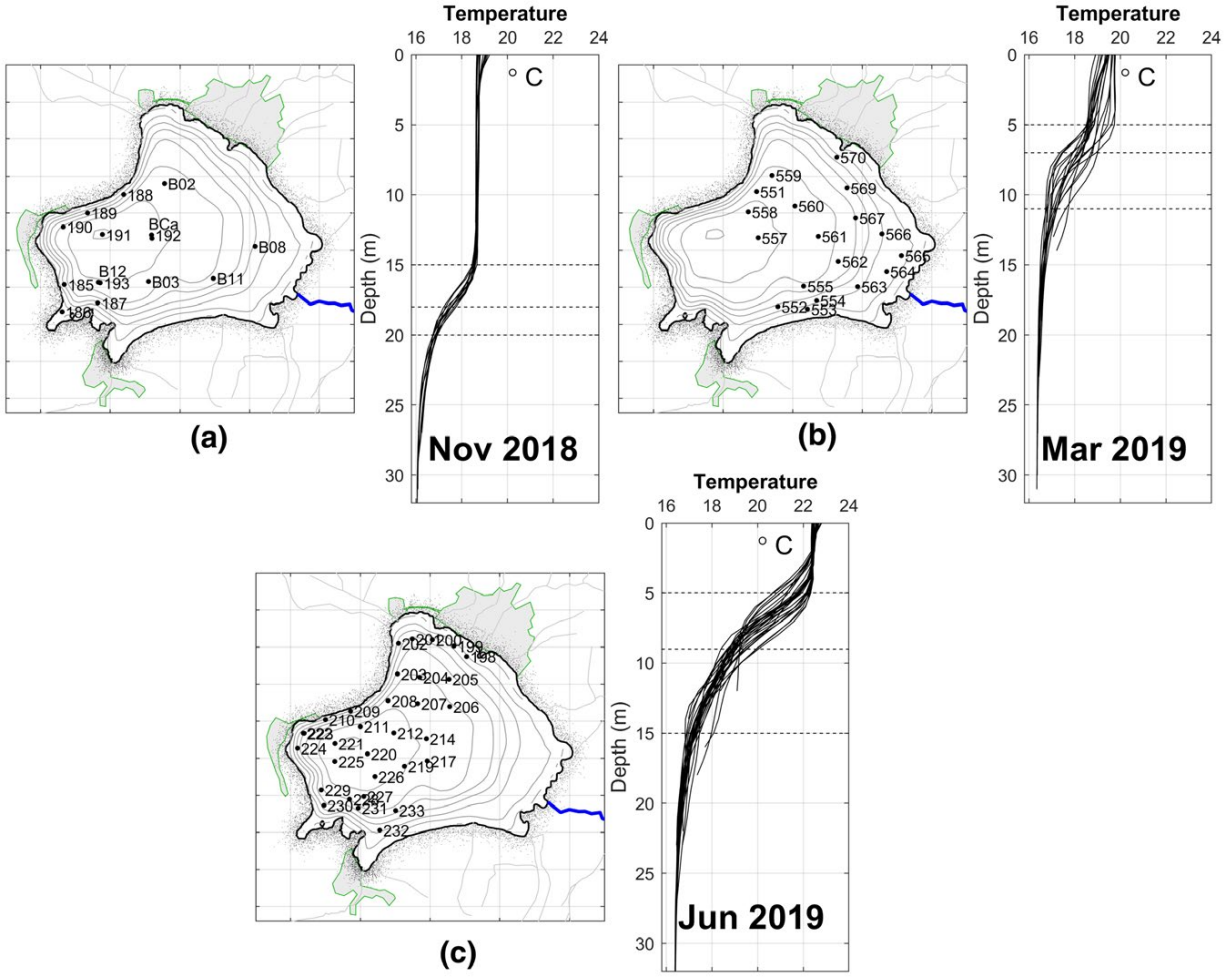
Location of CTD's profiles and thermal structure of Lake Zirahuén for (a) November 23, 2018, (b) March 19, 2019, and (c) June 19, 2019. The dashed lines correspond to the span of the thermocline

During the first campaign of November 23, 2018, 15 vertical profiles were made southwest of the lake with emphasis on the deeper zone (Figure 2a). A surface mixed layer with an average temperature of 18.6℃ was present and well developed to a depth of up to 15 m. The thermocline was in a narrow layer of no more than 5 m width and a depth of approximately 18 m. The hypolimnion existed from 20 m depth to the bottom, with an average temperature of 16.2℃. It is worth noting that the lake was going into its isothermal state.

For the second campaign on March 19, 2019, 18 profiles were carried out with emphasis on the northeast part of the lake near the shallower area and the river discharge (Figure 2b). A very shallow upper mixed layer appeared (5 m deep) with an average temperature of 19℃. The thermocline was located at approximately 7 m deep. In the hypolimnion zone (from 12 m), the average temperature was 16℃. In this time, a period of re‐stratification was underway.

During the third campaign of June 19, 2019, 32 profiles were made to the west of the lake with an emphasis on the deeper zone and near the principal locality of the lake (Figure 2c). The surface mixed layer had an average temperature of 22.2℃ and was well developed to a depth of up to 6 m, the thermocline was located at 9 m depth, and the hypolimnion presented an extension from 15 m to the bottom.

#### Chain's thermistors temperature

During early 2019, a heating in the lake was recorded showing an atypical behavior of the water column that took place around late December 2018 and stopped suddenly by the first days of February 2019, Figure 3. Although the thermistors were relatively deep, the temperature measured with the shallower sensor showed a rise of more than 0.5℃ during a very short time before January, and the deeper sensor data showed a rise of about 0.3℃, also in a very short time. Then, a fast decay occurred for the former sensor and the temperature for both subsequently remained practically constant until February, when they suddenly dropped by ~0.1℃. After this episode, the behavior of the time series remained typical for this lake, with a positive trend and a marked variability starting around May–June that shows the onset of the rainy seasonal period of the lake.

**FIGURE 3:**
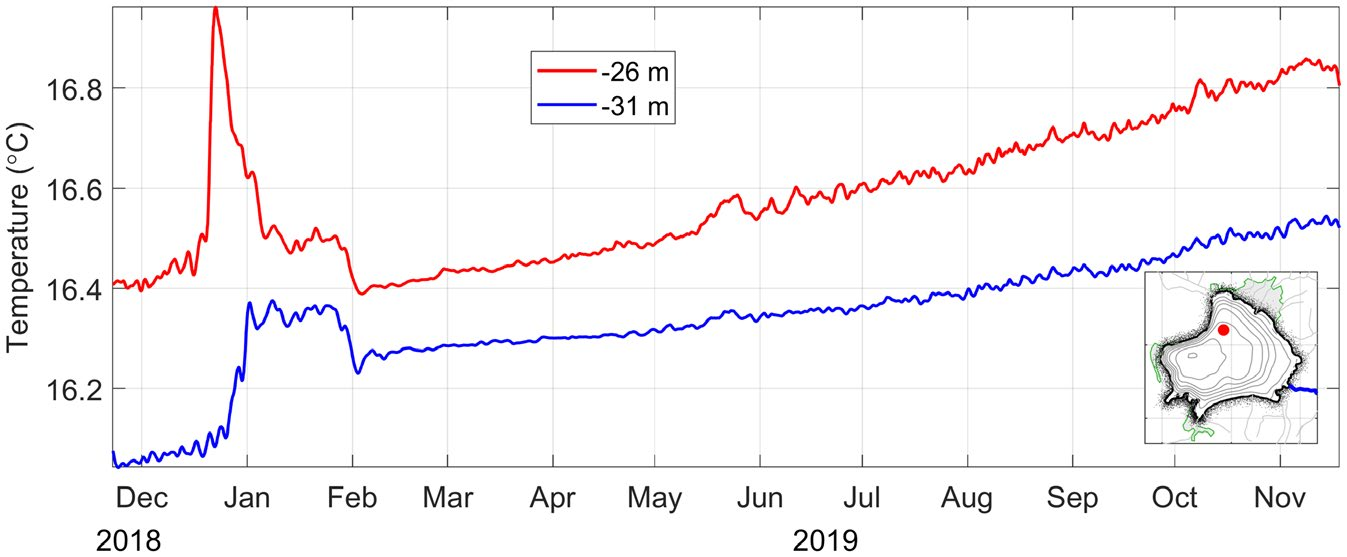
Temperature time series of two thermistors located at 26 and 31 m depth. Inset shows the position of the thermistor chain in the lake

### Trophic state

#### *Chl*‐*a for November 2018*

**FIGURE 4:**
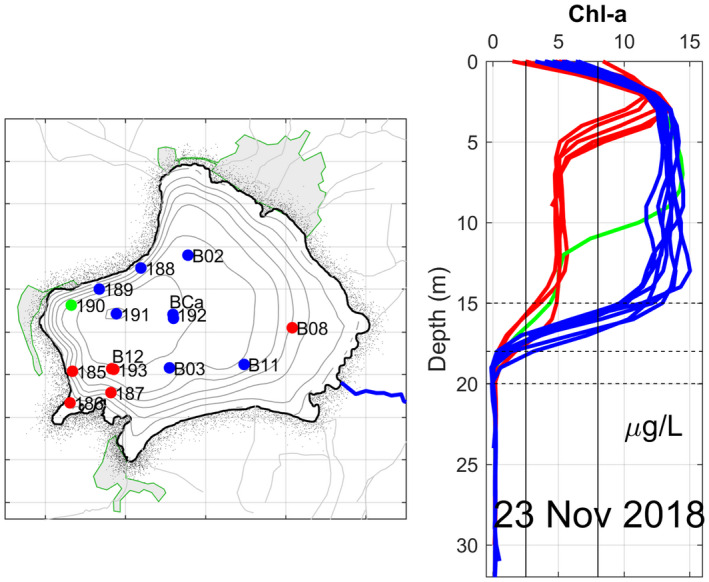
Location and vertical profiles of Chl‐a in Lake Zirahuén taken on November 23, 2018. In color are shown profiles with distinct characteristics. Vertical lines represent the limits of trophic state

During the campaign of 23 November, the highest concentration of Chl‐a was a subsurface value of 15.03 μg/L and was recorded at point B3, to the south of the lake (Figure 4). The profiles of this parameter show a characteristic behavior given by the stratification of the water column, that is, all of the maximum values (greater than 10 μg/L) are located above the thermocline and the minimum values (less than 4 μg/L) below the thermocline (see Figure 2a). At the surface, some spatial variability within all profiles is shown with values between 0 and 8 μg/L. Below, two types of behavior of great similarity can be observed for this parameter. Starting at ~3 m depth, the profiles that are located southwestward (for example, point 185) drop drastically down to 5 m depth, where they are then held constant for approximately another 10 m. Other profiles located in a more northeastward direction (such as B11) maintain a maximum value of Chl‐a close to 14 μg/L up to 15 m depth.

It is clear that all photosynthetic activity is found at a depth range of 0 to 15 m; in this range, Chl‐a values range between 4.5 μg/L and 14 μg/L. Between 15 and 18 m depth, Chl‐a values start to drop suddenly toward the bottom until dropping to practically zero.

The maximum Chl‐a value found for each profile is shown in Table 2, along with their respective trophic classification according to the OECD criterion for maximum Chl‐a (Gibson et al., 2000). As is verified, no spatial variation of the trophic state of the lake was found in the sampled area. All the profiles agree with the trophic classification that characterizes the lake in the mesotrophic range.

**TABLE 2:** Maximum values of chlorophyll‐a from November 23, 2018, and their respective trophic level according to the OECD classification

Points	Chl‐a (*μ*g/L) Maximum	Classification	Points	Chl‐a (*μ*g/L) Maximum	Classification
185	13.57	Mesotrophic	193	12.14	Mesotrophic
186	13.21	Mesotrophic	B02	13.93	Mesotrophic
187	12.47	Mesotrophic	B03	15.03	Mesotrophic
188	12.96	Mesotrophic	B08	12.99	Mesotrophic
189	14.51	Mesotrophic	B11	13.74	Mesotrophic
190	14.47	Mesotrophic	B12	11.90	Mesotrophic
191	14.34	Mesotrophic	BCa	13.70	Mesotrophic
192	13.44	Mesotrophic			

#### *Chl*‐*a and turbidity for March 2019*

**FIGURE 5:**
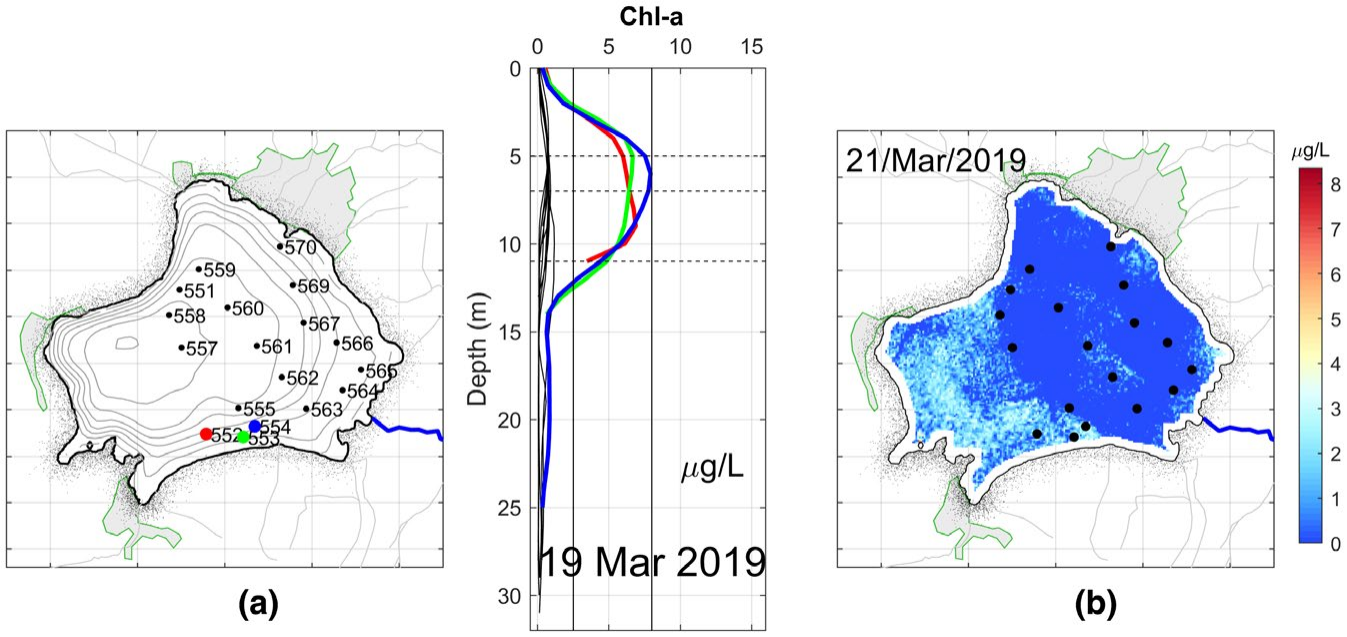
(a) Location and vertical CTD profiles of Chl‐a in Lake Zirahuén taken on 19 March 2019. In color are shown some profiles with distinct characteristics. (b) Spatial distribution of the estimated Chl‐a for March 21, 2019

The Chl‐a profiles obtained at each point on 19 March are shown in Figure 5a. In this case, the highest concentration of Chl‐a was 8.02 μg/L recorded at the subsurface at point 554. At the surface, the spatial variability was small with all of the profiles’ values near 0 μg/L.

Based on the Chl‐a estimated from satellite data, the spatial variability was reproduced adequately (Figure 5b). This figure was constructed based on a linear correlation (with a determination coefficient of *R* ^2^ = 0.64, see Supplementary materials) between the Chl‐a profiler data (Figure 5a) and the satellite data (the difference between reflectance bands B5 and B6). It is showing that the lake presents relatively higher values of Chl‐a at the southwestern section than at the northeastern section. Also, it is clearly seen that points 552, 553, and 554 were taken on the southern portion of the lake.

The maximum Chl‐a value found for each profile is shown in Table 3, along with their respective trophic classification. Again, the depth of the photic zone can be determined by observing the pattern or distribution of the Chl‐a profiles. Although not as equal as in November 2018, the higher maximum of Chl‐a is observed around 7 m depth, and then, it falls to a depth of between 9 and 11 m. Thus, the boundary of the photic zone is close to 11 m deep, less than that in November 2018 of 15 m.

**TABLE 3:** Maximum values of chlorophyll‐a from 19 March 2018 and their respective trophic level according to the OECD classification

Points	Chl‐a (*μ*g/L) Maximum	Classification	Points	Chl‐a (*μ*g/L) Maximum	Classification
551	1.17	Ultra‐Oligotrophic	561	0.77	Ultra‐Oligotrophic
552	6.88	Oligotrophic	562	0.78	Ultra‐Oligotrophic
553	8.07	Mesotrophic	563	0.85	Ultra‐Oligotrophic
554	8.02	Mesotrophic	564	0.91	Ultra‐Oligotrophic
555	0.74	Ultra‐Oligotrophic	565	0.71	Ultra‐Oligotrophic
557	0.70	Ultra‐Oligotrophic	566	0.82	Ultra‐Oligotrophic
558	0.72	Ultra‐Oligotrophic	567	0.75	Ultra‐Oligotrophic
559	0.68	Ultra‐Oligotrophic	569	0.67	Ultra‐Oligotrophic
560	0.78	Ultra‐Oligotrophic	570	0.64	Ultra‐Oligotrophic

As can be seen, large variations of Chl‐a were found and, as a result, variations in the trophic state. Although most of these values fall under the ultra‐oligotrophic classification, one point is considered as oligotrophic and two points are classified as mesotrophic. These three anomalous values were found in the same area, at the southeastern part of the lake.

**FIGURE 6:**
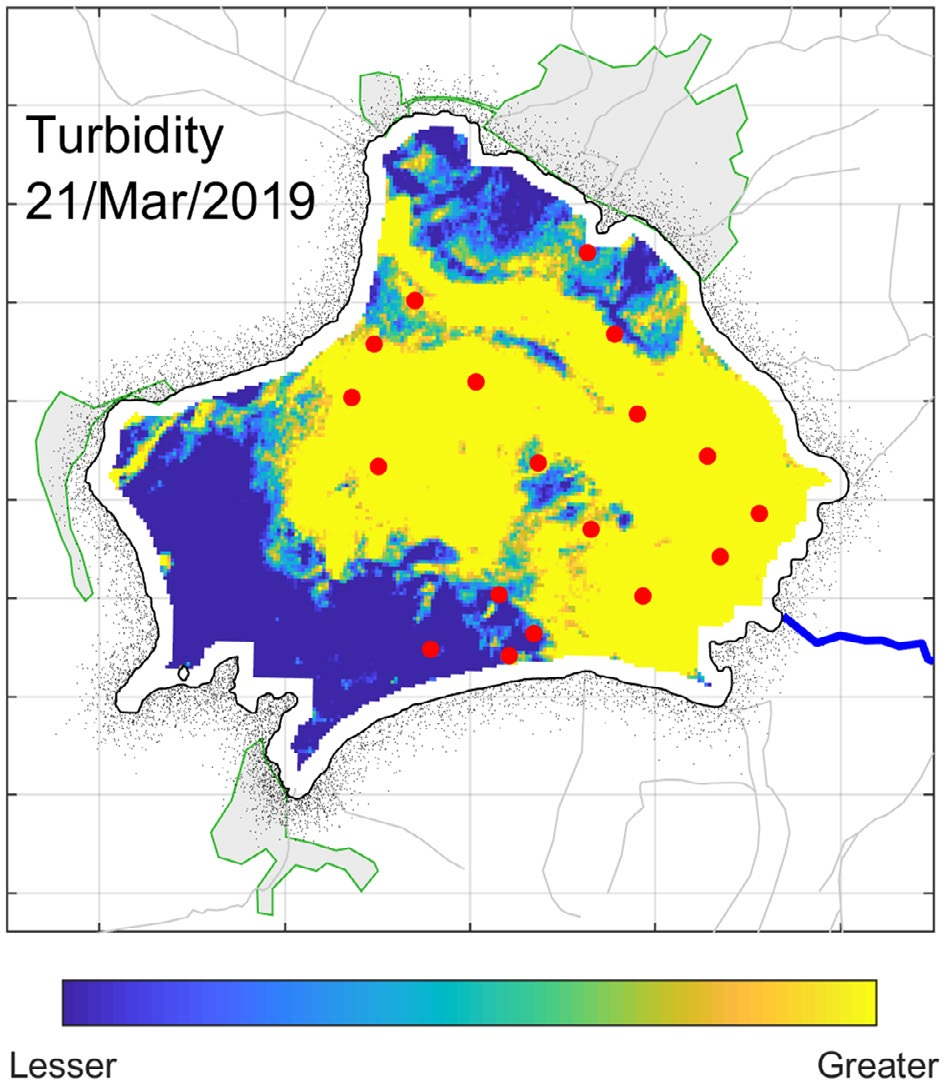
Reflectance of the B4 band uses a proxy for (qualitative) turbidity in Lake Zirahuén on the March 21, 2019. The yellow color indicates more turbidity (less clarity) and the blue color indicates less turbidity (more clarity). The red dots correspond to the location of the profiles

As mentioned above, turbidity, as an indirect measure of transparency, can be interpreted based on the reflectance of band B4. Unfortunately, transparency measurements were not taken, and however, highly useful qualitative information can be extracted from the satellite images (Figure 6), where it is evident the correlation with the spatial variability of the measured Chl‐a, especially at points 552 to 554. The spatial variability of the turbidity shows that the southwestern area of the lake was clearer than the rest of the lake. Then, the blocking of sunlight is preventing the passage to the lower levels in the northeastern then dramatically reducing photosynthetic activity below the surface layer.

#### *Chl*‐*a for June 2019*

**FIGURE 7:**
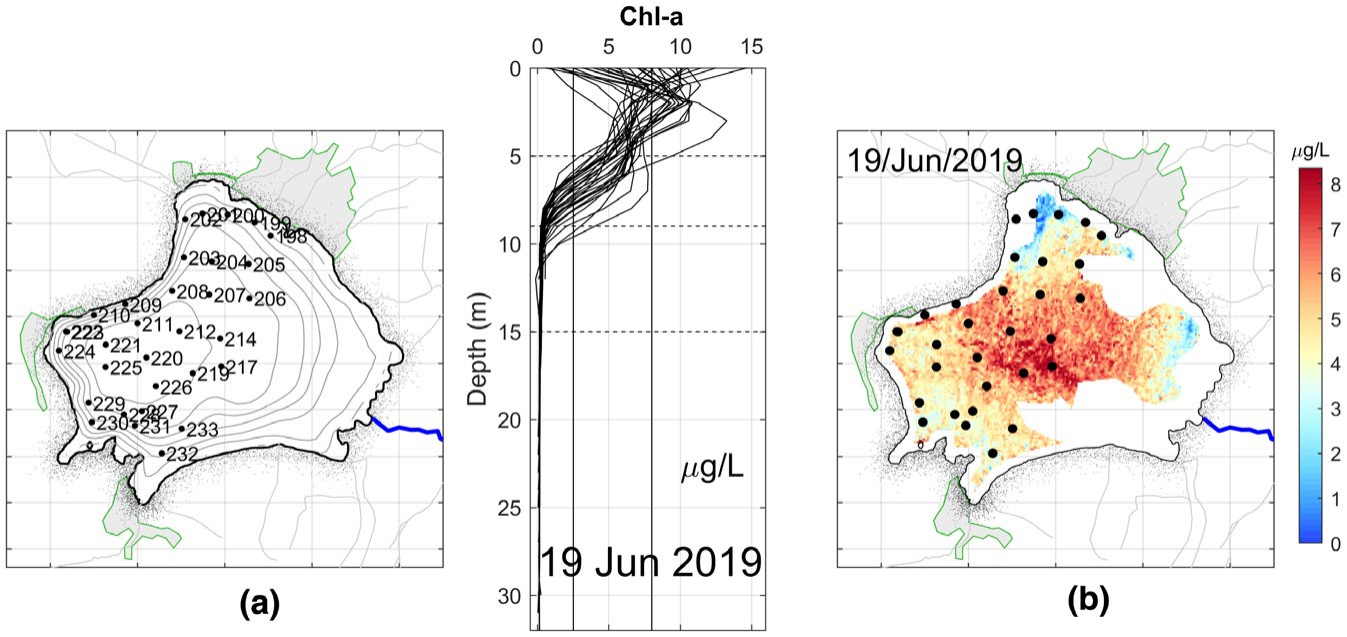
(a) Location and vertical profiles of Chl‐a in Lake Zirahuén taken on June 19, 2019. (b) Spatial distribution of the estimated Chl‐a for June 19, 2019

Figure 7a shows that on June 19, 2019, there was significant variability of Chl‐a at the surface, ranging from 0 to 15 μg/L. At this time, it was found that most of the maxima occur between the surface and 4 m depth. The Chl‐a values were insignificant below a depth of between 8 and 9 m. Thus, the depth of the photic zone was located close to 8 m, a depth even less than in March. The estimated Chl‐a from satellite data are shown in Figure 7b.

In this campaign, the maximum concentration of Chl‐a was 14.65 μg/L, which was recorded at point 225 near the deeper zone. The maximum Chl‐a value found for each profile is shown in Table 4, together with their respective trophic classification. It can be seen that in the vast majority of the points in the sampled area the trophic state was found to be mesotrophic.

**TABLE 4:** Maximum values of chlorophyll‐a from June 19, 2019 and their respective trophic level according to the OECD classification

Points	Chl‐a (*μ*g/L) Maximum	Classification	Points	Chl‐a (*μ*g/L) Maximum	Classification
198	10.52	Mesotrophic	217	9.26	Mesotrophic
199	11.48	Mesotrophic	219	10.56	Mesotrophic
200	10.28	Mesotrophic	220	10.25	Mesotrophic
201	10.10	Mesotrophic	221	9.65	Mesotrophic
202	5.75	Oligotrophic	222	10.07	Mesotrophic
203	6.31	Oligotrophic	223	10.78	Mesotrophic
204	7.74	Oligotrophic	224	13.26	Mesotrophic
205	11.42	Mesotrophic	225	14.65	Mesotrophic
206	7.55	Oligotrophic	226	7.65	Oligotrophic
207	12.50	Mesotrophic	227	9.91	Mesotrophic
208	8.15	Mesotrophic	228	9.23	Mesotrophic
209	7.27	Oligotrophic	229	10.63	Mesotrophic
210	10.23	Mesotrophic	230	9.37	Mesotrophic
211	8.95	Mesotrophic	231	7.06	Oligotrophic
212	10.73	Mesotrophic	232	10.86	Mesotrophic
214	7.76	Oligotrophic	233	8.02	Mesotrophic

#### **Sentinel‐2** **MSI, True Color Images, and the green band reflectance**

**FIGURE 8:**
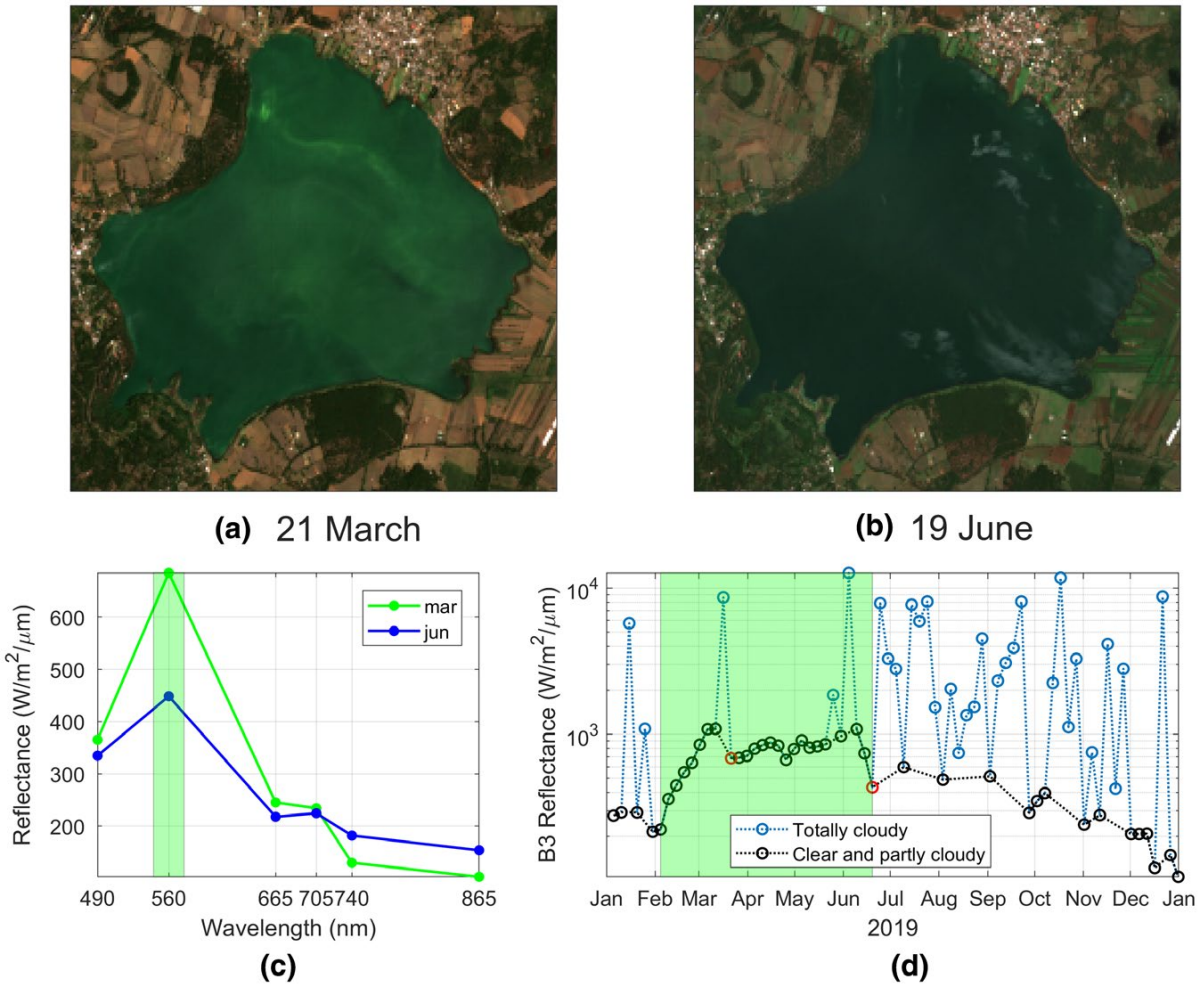
True Color Imageries (TCI) in Lake Zirahuén on (a) March 21, 2019, and (b) June 19, 2019. (c) Reflectance values on March 21, 2019, (green line) and June 19, 2019 (blue line), the $\lambda_{B3}=560\;\mathrm{nm}$ reflectance band is marked in green shading. (d) Time series of B3 reflectivity for ‘Clear and partly cloudy’ (black circles) and ‘Totally cloudy’ (blue circles) Sentinel MSI images. The green shaded region marks the period of an algal bloom in Lake Zirahuén. The red circles correspond to the values shown in (a) and (b); note the log scale on the y‐axis

True color images (TCI) are satellite products that have the function of presenting the target area as it is seen with the naked eye based on the B2, B3, and B4 spectral bands (blue, green, and red), respectively. In particular, the TCI for March and June present a lake with a green tone (Figure 8a,b), which is also quantified in the spectral signature of every image (Figure 8c). It is shown that at $\lambda_{B3}=560\;\mathrm{nm}$ the reflectance value of March is larger than for June, whereas at the other spectral bands are relatively in the same order. Note that the reflectance values are only considered within the lake even though the land surrounding the lake is getting greener by the growing vegetation due to the rainy season.

This anomalous behavior on the lake's color can be used as a proxy for tracking throughout the year the growth of vegetation within the lake (i.e., a eutrophication process), as shown in Figure 8d, where a time series of B3 reflectivity is created. It is shown that before the March campaign (first red circle) the lake went through a process that increase considerably the color on the lake generating what could be a possible algal bloom event. Then, during the June campaign (second red circle) the color on the lake diminished again to a more natural aspect of the lake but not as much as shown in Figure 1b.

These aspects are discussed in the following section in the context of the algal bloom event that took place at the beginning of the year and apparently was detect clearly by the satellite imagery.

## DISCUSSION

### Chl‐a and previous studies

Before going further into the algal bloom event, this section begins with some considerable differences between the observed Chl‐a values obtained during the campaigns of this study compared with the values recorded in previous studies of the lake (Table 5).

**TABLE 5:** Chl‐a values of previous studies in Lake Zirahuén and the values of this work

	Martínez‐Almeida and Tavera	Rendón et al.	Vergara et al.	Nov 2018	Mar 2019	Jun 2019
Max Chl‐a (µg/L)	3.98	2.8	1.42	15.03	8.07	14.65

These differences may be due to both the measurement method and the equipment used. Rendón et al., (2010) and Vergara de Paz et al., (2010) took samples for the determination of Chl‐a at only three different depths, whereas Martínez‐Almeida and Tavera (2005) took a sample every meter within the metalimnion and every 5 m in the remainder of the water column. All of these authors extracted the Chl‐a pigment and made their determination using fluorescence spectrophotometry. In contrast, for this study, a sensor profiler was used to measure this parameter in situ, performing a measurement every second and at a depth of approximately every meter. Thus, by taking more points in the water column, it is significantly more likely to find a maximum at each point using the profiler. Hence, a possible overestimation of the Chl‐a values can result from interference caused by other substances that fluoresce at the same wavelength as Chl‐a. As a result, this measurement method is usually used only for qualitative purposes to determine the distribution or trend of this parameter (Simpson & Sharples, 2012).

However, it cannot be ruled out that the higher concentration of this pigment found compared to those recorded in previous studies is due to a true increase in the degree of eutrophication of the lake, that is, a deterioration of its waters due to both natural and anthropogenic processes.

### Transparency

As already mentioned, it was not possible to perform transparency measurements. However, as a way to validate the trophic classification found above, it is possible to use the transparency values of the lake from previous studies and calculate an average to obtain a classification of the trophic state of the lake. Then, according to the average values of transparency from several studies and the OECD criteria, Lake Zirahuén is classified as mesotrophic (Table 6), which coincides with results of Tables 2 and 4 but not with those of Table 3. As can be seen, the values of transparency are very homogeneous, and then, it is expected that during the campaign of March 2019 an extraordinary event has happened, as will be seen in the next section.

**TABLE 6:** Secchi transparency values taken from previous studies in Zirahuén

Secchi transparency (m)	Rendón et al.	Israde et al.,	Vergara et al.	Madrigal and Chacón	Averages
Medium	4.0	3.4	3.6	‐	3.6
Minimum	2.0	1.2	2.6	2.1	2.0
Maximum	6.0	5.0	5.7	4.6	5.3

### The algal bloom event

The observations taken during March were for most the more remarkable campaign given that the lake is considered in the range of oligotrophic‐mesotrophic level. In the previous and after campaigns, no significant spatial variations on the trophic state were found, so it is thought that the higher variability was due to the layer of suspended organic matter covering the lake. During this campaign, the lake was covered by a dense layer of organic matter because of the excessive proliferation of algae (Figure 1c), which, as known from direct communication with local people, began a few days before this campaign, and was possibly triggered by the last rains following the winter season. Much of the land surrounding Lake Zirahuén is used for agriculture and for animal grazing (due to a recent change in land use). Because of these activities, the soil accumulates a large amount of nutrients, especially during the dry season when the rain does not periodically infiltrate the nutrients into the soil. As a result, during the early rains of the year, the lake manifests an excessive proliferation of algae due to the accumulated nutrients carried to the lake, which increases the photosynthesis reaction and, thus, the production of algae. These algae agglomerate in large quantities near the surface of the water, blocking sunlight and then preventing its passage to the lower levels of the water column, dramatically reducing photosynthetic activity below the surface layer. This explains the low levels of Chl‐a registered during the campaign of March 2019 compared to those measured in November 2018 and June 2019.

Although this might seem inconsistent (and even paradoxical) since eutrophication means excess of nutrient and hence higher values of Chl‐a, the results are showing the opposite, that is, lower values of Chl‐a during a clearly visual confirmation that the lake was undergoing an algal bloom (Figures 5 and 1c). However, this can be explained using the time series of Figure 8d.

It can be said that the algal bloom started around early February and remained until around mid‐June (green shading in Figure 8d), showing a positive trend in the color of the lake during the whole month of February, which is also indicative of algae growth. Then, after decreasing considerably precisely during the time of the in situ observation in March, the color was going into a series of alternating positive and negative trends. It is during this time that the algae were already decomposing (and start to naturally change color) blocking light effects resulting in the yellowish color of the lake and hence the result of the low values of Chl‐a.

Based again in Figure 8d, it is not possible to determine the day of June or July during which the lake recovered its normal state due to the increasing reflectance in all bands caused by the rainy season, which resulted in cloudy images for most of the days between June and December (blue circles in Figure 8d). However, a risky assumption can be made about the relationship between a cloudy sky and the occurrence of rain, that is, it can be inferred that two possible events of precipitation occurred during January which may have carried the nutrients from the surrounding agricultural land into the lake, hence triggering the algal bloom. Unfortunately, we do not have measurements from a weather station during this time to confirm this. Then, if the rain took place and it was combined with the heating of the lake that occurs throughout the month of January (Figure 3), this undoubtedly created the perfect conditions for the algal bloom during March. Those two factors were for much the principal ingredients to develop such an event. Regrettably, the nutrient input was surely carried by fertilizer runoff from the agricultural lands surrounding the lake, whereas the warming event was triggered by mixing caused by the typical valley‐mountain breeze that blows over this area.

## CONCLUSIONS

Spatial/temporal variations exist in the trophic state of Lake Zirahuén because the same trophic status classification was not obtained for all the campaigns undertaken in this study. The lake is classified as being in transition from oligotrophic to mesotrophic. However, as the majority of the profile points fell into the mesotrophic classification, the lake is more closely aligned with this category. Nonetheless, it continues to have good clarity.

With respect to the satellite images, it was found an improvement in the description of the spatial‐temporal variability of the lake. In particular, the turbidity and the time series of the green bands were highly useful for describing the algal bloom that peaked around March 2019, and then, it is believed that this analysis is an adequate tool that can be used in more efficient ways to monitor lakes.

## CONFLICTS OF INTEREST

The authors declare no conflict of interest.

## AUTHOR CONTRIBUTION

**Diego A Pantoja:** Formal analysis (equal); Funding acquisition (lead); Investigation (equal); Project administration (lead); Supervision (equal); Writing‐original draft (equal). **Néstor Alberto Vega‐Álvarez:** Conceptualization (equal); Investigation (equal); Writing‐original draft (equal). **Tzitlali Gasca‐Ortiz:** Conceptualization (equal); Formal analysis (equal); Investigation (equal); Methodology (equal); Writing‐review & editing (equal).

## Supporting information

Appendix S1
