# Supplementary material for: Trophic state in a tropical lake based on Chlorophyll‐a profiler data and Sentinel‐2 images: The onset of an algal bloom event
Source: Water Environ Res. 2021 Jun 22;93(10):2185–97. doi: 10.1002/wer.1590 (PMC8596837; doi:10.1002/wer.1590)
Supplement: Supplementary file 1 — Appendix S1 [file WER-93-2185-s001.docx]

**Supplementary materials**

**Appendix A. Sentinel-2 MSI imagery**

The concentration of Chl-a was estimated based on the empirical equations that combines reflectance from different bands (Grendaitė et al., 2018; Peppa et al., 2020). In Table A1, are shown some indices tested in this study.

Table A1. Indices for estimation of Chl-a and turbidity

| Equation for Chl-a |
| --- |
| $MCI=B5-B4-\frac{(\lambda_{B5}-\lambda_{B4})}{(\lambda_{B6}-\lambda_{B4})}(B6-B4)$ |
| $MPH=B5-B4-\frac{\left( \lambda_{B5}-\lambda_{B4} \right)}{\left( \lambda_{B8A}-\lambda_{B4} \right)}(B8A-B4)$ |
| $Chla\_1=B5$ |
| $Chla\_2=B5-\frac{1}{2}(B4-B6)$ |
| $Chla\_3=B5-B6$ |
| $Chla\_4=\frac{B8A}{B4}$ |

The maximum chlorophyll index (MCI) is one of eutrophic indicators most used based on the reflectance peak at $\lambda_{B5}=705 nm$ of the electromagnetic spectrum, which is associated with high levels of Chl-a and hence the eutrophication of the basin waters. B4, B5, and B6 are the bands of reflectance at $\lambda_{B4}=665 nm$, $\lambda_{B5}=705 nm$, and $\lambda_{B6}=740 nm$ central wavelengths, respectively. This index weight the height of the peak at the B5 band against the baseline of its adjacent peaks at the B4 and B6 spectral bands.

Other indices were tested, for example, the maximum peak-height (MPH) commonly used for cyanobacteria blooms (Grendaitė et al., 2018; Toming et al., 2016), with B8A the reflectance band at $\lambda_{B8A}=865 nm$. However, less elaborated indices (Chla’s of Table A1) based on B5 or B8A reflectance bands, result more appropriate for the case of Lake Zirahuén (Cairo et al., 2020; Membrillo-Abad et al., 2016; Peppa et al., 2020).

Based on the Chl-a profiler data, the spatial variability and its relationship with the different indices of Table A1 were obtained in Figure A1. The selection of the most favorable index to obtain the best fit consisted in (i) qualitatively reproduce the correct spatial variability of the Chl-a and (ii) quantitatively, have a good agreement based on the R^2^, RMSE, and the Student t test values. Only data from Chl-a sensor of the profiler from 19 March and 19 June were used.

| 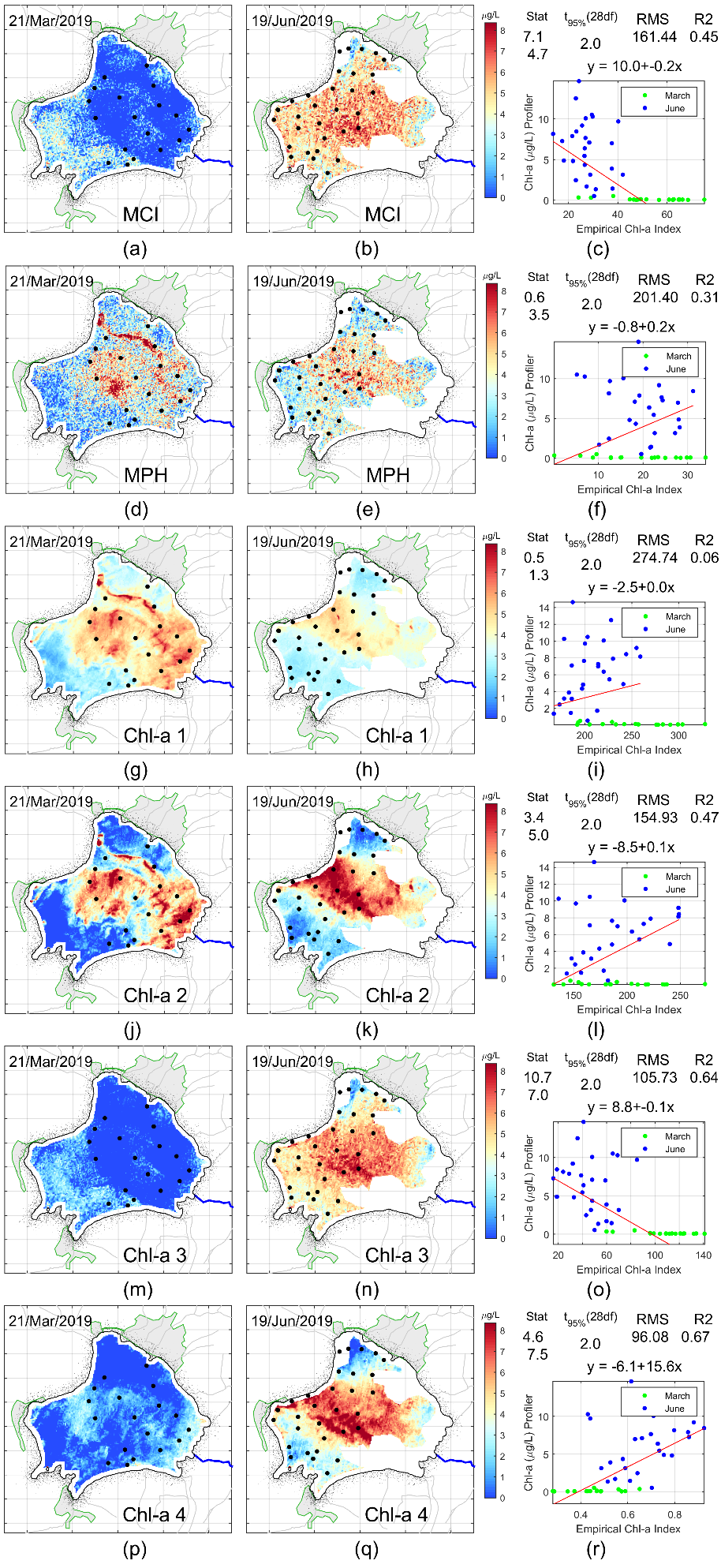 |
| --- |

Figure A1. Spatial distribution of Chl-a based on MCI for (a) March 21, 2019, (b) June 19, 2019, and (c) linear correlation with profiles data. Same in (d), (e), and (f) for MPH index. In (g), (h), and (i) for Chl-a_1 index. In (j), (k), and (l) for Chl-a_2 index. In (m), (n), and (o) for Chl-a_3 index. In (p), (q), and (r) same for Chl-a_4 index.

The MCI (Figure A1a,b,c) as the prime index to retrieve Chl-a from satellite imagery shows a spatial variability that is in high correspondence with the inferred description mention above (see Figures 5, 6, and 8). In particular, higher values of Chl-a were found at the southern section of the lake during March (Figure A1a), and higher values across the lake during June (Figure A1b), except at the northern section. Qualitatively, the spatial variability shows some granulated aspect meanwhile the best linear fit shows that both coefficients (the constant-term 10 and the linear-term -0.2) are in good agreement with a 95% confident level (since their corresponding statistical values: 7.1 and 4.7 are greater than 2.0 for 28 degrees of freedom (Laws, 1997)). The RMSE is relatively small as compared with other fits, and the determination coefficient R^2^ that is also small (< 0.5). Nonetheless, the imageries show good performance.

The MPH index (Figure A1d,e,f) and the Chl-a_1 index (Figure A1g,h,i) and Chl-a_2 index (Figure A1j,k,l), even though the June outputs show an improvement in the clearness, the results are not satisfactory compared to March outputs against the MCI output. The Chl-a should be smaller at the center of the lake, and in fact, it is in inverted order. The analytical adjust also shows a poor performance as the RMSE is larger and the coefficient of determination R^2^ is smaller for the case of the MPH index and the Chl-a_1 index. Even the coefficients of the linear fit are not completely confident at 95% since the statistical value of 0.6 is not > 2.0 for MPH; and 0.5 and 1.3 are not > 2.0 for Chl-a_1. Although the Chl-a_2 index shows an improvement respect to the other two, qualitative and analytically, its spatial variability remains inverted for March.

The Chl-a_3 index (Figure A1n,m,o) and Chl-a_4 index (Figure A1p,q,r) fit improved compared with MCI having the RMSE relatively smaller and R^2^ higher. Despite the better analytical performance of Chl-a_4 index against the Chl-a_3 index, the spatial variability of the latter resembles more the MCI behavior, but with clearer resolution, so for this case, the Chl-a_3 index is the better option for Lake Zirahuén.
